# C-reactive protein and statins in heart failure with reduced and preserved ejection fraction

**DOI:** 10.3389/fcvm.2022.1064967

**Published:** 2022-12-23

**Authors:** Jin Joo Park, Minjae Yoon, Hyoung-Won Cho, Hyun-Jai Cho, Kye Hun Kim, Dong Heon Yang, Byung-Su Yoo, Seok-Min Kang, Sang Hong Baek, Eun-Seok Jeon, Jae-Joong Kim, Myeong-Chan Cho, Shung Chull Chae, Byung-Hee Oh, Dong-Ju Choi

**Affiliations:** ^1^Department of Internal Medicine, Seoul National University College of Medicine, Seoul National University Bundang Hospital, Seongnam, Republic of Korea; ^2^Department of Internal Medicine, Yonsei University College of Medicine, Seoul, Republic of Korea; ^3^Department of Internal Medicine, Seoul National University Hospital, Seoul, Republic of Korea; ^4^Heart Research Center, Chonnam National University, Gwangju, Republic of Korea; ^5^Department of Internal Medicine, Kyungpook National University College of Medicine, Daegu, Republic of Korea; ^6^Department of Internal Medicine, Yonsei University Wonju College of Medicine, Wonju, Republic of Korea; ^7^Department of Internal Medicine, The Catholic University of Korea, Seoul, Republic of Korea; ^8^Department of Internal Medicine, Sungkyunkwan University College of Medicine, Seoul, Republic of Korea; ^9^Department of Internal Medicine, University of Ulsan College of Medicine, Seoul, Republic of Korea; ^10^Department of Internal Medicine, Chungbuk National University College of Medicine, Cheongju, Republic of Korea; ^11^Division of Cardiology, Department of Internal Medicine, Kyungpook National University Chilgok Hospital, Daegu, Republic of Korea; ^12^Division of Cardiology, Cardiovascular Center, Incheon Sejong Hospital, Incheon, Republic of Korea

**Keywords:** heart failure, inflammation, outcomes, C-reactive protein, statin

## Abstract

**Background:**

High C-reactive protein (CRP) levels are associated with poor outcomes of heart failure (HF), and statins are known to reduce CRP levels. We investigated the prognostic value of CRP and statin in patients with HF with reduced and preserved ejection fraction (EF).

**Methods:**

Altogether, 3,831 patients from the Korean Acute Heart Failure registry were included and stratified according to the tertiles of CRP levels (T1: CRP < 0.30 mg/dL, T2: 0.30–1.14 mg/dL, and T3: CRP > 1.14 mg/dL). HF with reduced EF (HFrEF), HF with mildly reduced EF (HFmrEF), and HF with preserved EF (HFpEF) were defined as left ventricular ejection fraction (LVEF) ≤ 40%, 41–49%, ≥50%, respectively. The primary endpoints were all-cause, in-hospital, and post-discharge mortality.

**Results:**

No significant correlation was observed between CRP levels and LVEF (*r* = 0.02, *P* = 0.131). The prevalence of risk factors increased gradually from T1 to T3 in both the types of HF. Overall, 139 (3.6%) and 1,269 (34.4%) patients died during the index admission and follow-up (median: 995 days), respectively. After adjustment, each increase in the CRP tertiles was independently associated with in-hospital mortality (HFrEF: OR 1.58 and 95% CI 1.09–2.30, HFmrEF: OR 1.51 and 95% CI 0.72–3.52, and HFpEF: OR 2.98, 95% CI 1.46–6.73) and post-discharge mortality (HFrEF: HR 1.20, 95% CI 1.08–1.33, HFmrEF: HR 1.38 and 95% CI 1.12–1.70, and HFpEF: HR 1.37, 95% CI 1.02–1.85). In only patients with LVEF > 40% with highest CRP tertile, statin-users showed better survival trend than those without statins.

**Conclusion:**

CRP is an excellent prognostic marker for HFrEF, HFmrEF, and HFpEF, implying that the neurohumoral and inflammatory pathways might be independent pathways. Statins may be beneficial in HF patients with increased CRP levels.

**Clinical trial registration:**

[https://clinicaltrials.gov/], identifier [NCT013 89843].

## Introduction

Neurohumoral (1–5), inflammatory (6–8), and cardiometabolic pathways (9) play an important role in the progression of heart failure (HF). HF is classified into two types according to the ejection fraction (EF): HF with reduced EF (HFrEF) and HF with preserved EF (HFpEF). Due to the differences in cardiac anatomy, patients with HFrEF exhibit higher b-type natriuretic peptide (BNP) levels than those with HFpEF, reflecting a higher degree of neurohumoral activity (10, 11). Thus, drugs targeting the neurohumoral pathways such as renin-angiotensin system (RAS) inhibitors (including angiotensin converting enzyme inhibitors), beta-blockers, and mineralocorticoid receptor antagonists (MRAs) are effective in HFrEF, but not in HFpEF (12). Regarding the inflammatory pathways, HFpEF had higher levels of inflammatory biomarkers than HFrEF (13–16). In contrast, the metabolic pathways may be independent of left ventricular EF (LVEF). Indeed, sodium-glucose co-transporter 2 (SGLT2) inhibitors have shown improved clinical outcomes in patients with HFrEF (17, 18) as well as in those with HFpEF (19).

Among different inflammatory markers, C-reactive protein (CRP) is the most investigated marker and has been identified as a strong risk factor for the development and progression of HF (20–22). However, few studies focused on the differential effects of inflammatory markers according to HF phenotypes showed inconsistent results (14–16, 23). Statins have been shown to reduce CRP levels and vascular events in patients with elevated CRP levels (24). However, statins did not improve cardiovascular mortality in patients with HF (25, 26).

We hypothesized that the prognostic impact of CRP is independent of HF phenotypes and patients with elevated CRP levels may benefit from statins. Therefore, we investigated the differential effect of CRP on clinical outcomes according to the type of HF in Korean patients admitted for acute HF. Subsequently, we investigated the differential effects of statins on post-discharge survival according to CRP levels.

## Materials and methods

### Study design and population

The Korean Acute Heart Failure (KorAHF) registry was a prospective multicenter cohort study that consecutively enrolled 5,625 patients hospitalized for acute HF syndrome in 10 tertiary university hospitals throughout the country between March 2011 and December 2014. Detailed information on the study design and results has been reported previously (ClinicalTrials.gov NCT01389843) (27, 28). Briefly, patients who had signs or symptoms of HF and met one of the following criteria were eligible for this study: (1) lung congestion or (2) objective left ventricular systolic dysfunction or structural heart disease findings. For assessment of structural heart disease chamber sizes and wall thickness, valve anatomy and functions, presence of cardiomyopathies, and for assessment of diastolic dysfunction left atrial volume index, e′ velocity, and E/e′, among others, were evaluated during the echocardiography by the attending physicians. In the present study, we only included patients whose acute decompensation was not triggered by infection and those with availability of data regarding CRP levels or LVEF. Regarding the aggravating factor for acute decompensation, the responsible physician was asked to choose one of the following factors as the most-likely trigger for acute decompensation, which included acute coronary syndrome, severe hypertension, atrial or ventricular tachyarrhythmia, bradycardia, infection, pulmonary emboli, renal failure, anemia/bleeding, medication (e.g., NSAIDs), non-compliance, endocrinal abnormality, and recent addition of negative inotropic agents. The information on the aggravating factor was prospectively collected and was adjudicated before discharge by the investigators (28).

The study protocol was approved by the Ethics Committee and institutional review board (IRB) of each hospital (Seoul National University Bundang Hospital, IRB No. B-1104-125-014). The study was conducted in accordance with the Declaration of Helsinki and all patients provided written informed consent upon enrollment.

### Data collection and study endpoints

All echocardiographic studies were performed by cardiologists who were certified by Korean Society of Echocardiography, using a standard ultrasound machine with a 2.5-MHz probe. Standard techniques were adopted to obtain M-mode, 2-dimensional, and Doppler measurements in accordance with the American Society of Echocardiography’s guidelines (29). LVEF was measured using Simpson’s biplane method, unless Simpson’s method was not possible. Most patients underwent echocardiography on the day of the admission (the median time interval between admission and echocardiographic exam was 1 day with an interquartile range of 0–2 days). Based on echocardiography findings, HFrEF, HF with mildly reduced EF (HFmrEF) and HFpEF were defined as LVEF ≤ 40%, 41–49%, and ≥50%, respectively (12). Routine blood sampling and tests were conducted by laboratories at each center that were certified by the Korean Association of Quality Assurance for Clinical Laboratory. CRP levels were measured at the index admission. CRP and high-sensitivity CRP levels were measured using a high-sensitivity immunoturbidimetric method. Patients were classified according to the type of HF and CRP tertiles: first tertile [T1]: CRP < 0.30 mg/dL, T2: CRP level 0.30–1.14 mg/dL, T3: CRP > 1.14 mg/dL. The use of statins was evaluated at hospital discharge. The primary endpoints were all-cause, in-hospital, and post-discharge mortality. The mortality data of patients who were lost to follow-up were collected from the Korean Statistical Information Service and Microdata Integrated Service managed by Statistics Korea, a government agency.

### Statistical analysis

Data are presented as numbers and frequencies for categorical variables and as means ± standard deviation for continuous variables. For comparison among groups, the χ^2^ test (or Fisher’s exact test when any expected count was <5 for a 2 × 2 table) was applied for categorical variables and the unpaired Student’s *t*-test or one-way analysis of variance was applied for continuous variables.

A receiver operating characteristic (ROC) curve was obtained to compare the prognostic performance of CRP in both the types of HF. The Get With The Guidelines-Heart Failure (GWTG-HF) score was calculated for each patient (30) to estimate the risk. The relationship between CRP and other variable was evaluated using Pearson correlation coefficient. Kaplan–Meier curves were plotted and compared using the log-rank test for the evaluation of post-discharge outcomes. Multivariable logistic regression and Cox proportional hazards regression models were used to determine the independent effects of CRP levels on in-hospital and post-discharge outcomes, respectively. We adjusted for variables associated with mortality except variables with >10% missing values or variables that were closely related to other clinical variables. Thus, we adjusted for age, sex, new-onset HF, diabetes, ischemic heart disease, chronic obstructive pulmonary disease, cerebrovascular disease, New York Heart Association class, systolic blood pressure, heart rate, white blood cell (WBC) count, hemoglobin, blood urea nitrogen, E/e′, LVEF, CRP tertiles, and the use of RAS inhibitors, beta-blockers, and MRAs. Since GWTG-HF score stratify the risk of patients, we developed models with adjustment for the GWTG-HF score as a sensitivity analysis.

Statistical significance was set at a two-sided *P*-value < 0.05. Statistical analyses were performed using SPSS version 25.0 (IBM Corp., Armonk, NY, USA) and R programming version 4.0.2 (The R Foundation for Statistical Computing, Vienna, Austria).

## Results

A total of 5,625 patients were enrolled in the KorAHF registry. Among these, acute decompensation was triggered by infection in 1,102 (19.6%) patients and these patients exhibited higher CRP levels than their counterparts (4.23 ± 6.02 mg/dL vs. 1.88 ± 3.52 mg/dL, *P* < 0.001). In addition, 373 (6.6%) and 522 (9.3%) patients did not have data regarding CRP levels and LVEF, respectively. Thus, data from 3,831 patients were available for the final analysis. Supplementary Table 1 presented the baseline characteristics of patients included (*n* = 3,831) and excluded (*n* = 6,922) due to missing CRP levels or LVEF data. Although few variables such as *de novo* heart failure, estimated glomerular filtration rate < 60 ml/min/1.73 m^2^, smoking, NYHA class, blood pressure, and medications differ between included and excluded patients, baseline characteristics were overall comparable between the two groups. The mean age was 68.2 years, 53.9% were male, 58.8% of the patients had hypertension, and 35.0% of the patients had diabetes.

According to the definitions of the types of HF, 2,267, 559, and 1,005 patients had HFrEF, HFmrEF, and HFpEF, respectively, and they had similar CRP levels (HFrEF: 1.78 ± 3.31 mg/dL vs. HFmrEF: 2.09 ± 3.83 mg/dL vs. HFpEF: 1.95 ± 3.70 mg/dL, *P* = 0.133). No significant correlation was observed between the CRP levels and LVEF (*r* = 0.02, *P* = 0.131). In the ROC curve analysis, CRP level exhibited areas under the curve (AUCs) of 0.67 [95% confidence interval (CI) 0.62–0.73], 0.67 (95% CI 0.56–0.80), and 0.70 (95% CI 0.59–0.82) to predict in-hospital mortality for HFrEF, HFmrEF, and HFpEF, respectively. To predict post-discharge mortality, the AUCs of CRP were 0.59 (95% CI 0.57–0.62), 0.62 (95% CI 0.57–0.67), and 0.58 (95% CI 0.54–0.61) for HFrEF, HFmrEF, and HFpEF, respectively.

The patients were stratified and their clinical characteristics were presented according to the tertiles of CRP levels (Table 1). There was a gradual increase in the prevalence of adverse characteristics such as hypertension, diabetes, chronic kidney disease, and natriuretic peptide levels from T1 to T3.

**TABLE 1:** Baseline characteristics of the study population according to the heart failure (HF) categories and tertiles of C-reactive protein (CRP) levels.

	Overall (*n* = 3,831)				HFrEF (*n* = 2,267)				HFmrEF (*n* = 559)				HFpEF (*n* = 1,005)			
	T1 (*n* = 1,380)	T2 (*n* = 1,183)	T3 (*n* = 1,268)	*P*-value	T1 (n = 790)	T2 (*n* = 720)	T3 (*n* = 757)	*P*-value	T1 (*n* = 198)	T2 (*n* = 181)	T3 (*n* = 180)	*P*-value	T1 (*n* = 392)	T2 (*n* = 282)	T3 (*n* = 331)	*P*-value
Age (years)	67.2 ± 14.5	68.0 ± 14.2	69.3 ± 14.3	0.001	64.8 ± 15.1	66.0 ± 14.5	67.3 ± 14.5	0.003	70.6 ± 12.1	70.4 ± 13.6	71.4 ± 13.0	0.702	70.4 ± 13.5	71.9 ± 12.9	72.8 ± 14.0	0.054
Men (%)	49.7%	54.5%	57.8%	<0.001	57.7%	61.4%	65.8%	0.005	43.9%	50.3%	53.9%	0.145	36.5%	39.7%	41.7%	0.348
*De novo* (%)	53.9%	53.4%	59.2%	0.005	51.1%	52.8%	56.1%	0.134	59.6%	63.0%	62.2%	0.775	56.6%	48.9%	64.7%	<0.001
Body mass index (kg/m^2^)	23.5 ± 3.8	23.6 ± 3.9	23.1 ± 3.7	0.003	23.1 ± 3.8	23.4 ± 3.9	22.9 ± 3.8	0.016	23.4 ± 3.2	23.8 ± 4.0	23.3 ± 3.7	0.458	24.4 ± 4.2	23.7 ± 4.0	23.5 ± 3.5	0.004
**Past medical history**																
Hypertension (%)	56.2%	57.7%	62.7%	0.002	50.5%	55.4%	59.2%	0.003	62.6%	59.1%	74.4%	0.006	64.3%	62.4%	64.4%	0.853
Diabetes mellitus (%)	32.5%	33.3%	39.3%	<0.001	34.1%	35.8%	40.7%	0.021	34.8%	30.9%	41.1%	0.126	28.1%	28.4%	35.0%	0.085
eGFR < 60 ml/min/1.73 m^2^ (%)	36.7%	42.7%	51.8%	<0.001	36.5%	43.8%	54.6%	<0.001	40.4%	34.3%	50.0%	0.009	35.5%	45.4%	46.5%	0.004
Ischemic heart disease (%)	28.0%	25.4%	29.9%	0.043	27.6%	28.0%	33.6%	0.016	32.3%	27.6%	29.4%	0.600	26.5%	17.4%	21.8%	0.018
Atrial fibrillation (%)	24.9%	31.7%	24.2%	<0.001	21.3%	26.3%	22.1%	0.050	23.7%	38.7%	23.3%	0.001	32.7%	41.1%	29.6%	0.008
COPD (%)	9.9%	9.8%	12.1%	0.100	9.4%	8.6%	11.9%	0.086	12.1%	8.3%	10.6%	0.658	12.0%	13.8%	13.6%	0.731
Cerebrovascular disease (%)	11.8%	14.6%	18.1%	<0.001	10.3%	13.8%	19.0%	<0.001	12.1%	16.7%	15.0%	0.445	14.8%	15.2%	17.8%	0.506
Malignancy (%)	7.0%	7.4%	9.5%	0.033	6.8%	7.6%	10.7%	0.016	8.1%	8.3%	9.4%	0.881	6.6%	6.0%	6.9%	0.898
Current smoking (%)	16.5%	19.6%	19.3%	0.128	19.9%	23.5%	22.7%	0.205	9.6%	16.0%	17.2%	0.070	13.3%	12.1%	12.7%	0.897
NYHA functional class (%)				<0.001				<0.001				<0.001				<0.001
II	22.6%	15.0%	11.0%		19.4%	13.8%	9.5%		27.8%	17.7%	10.6%		26.5%	16.3%	14.5%	
III	39.1%	40.8%	29.9%		40.9%	40.7%	28.4%		35.4%	35.9%	32.8%		7.5%	44.3%	31.7%	
IV	38.3%	44.2%	59.1%		39.7%	45.6%	62.1%		36.9%	46.4%	56.7%		36.0%	39.4%	53.8%	
**Physical Exam**																
Systolic BP (mmHg)	130.7 ± 28.7	133.6 ± 29.6	131.0 ± 31.3	0.029	127.9 ± 27.6	131.1 ± 28.2	127.5 ± 30.1	0.032	134.7 ± 30.5	139.5 ± 30.8	136.2 ± 34.8	0.340	134.5 ± 29.3	136.4 ± 31.6	136.0 ± 31.2	0.699
Diastolic BP (mmHg)	77.9 ± 18.8	81.6 ± 18.8	77.5 ± 19.2	<0.001	78.1 ± 17.6	82.3 ± 18.5	78.4 ± 18.9	<0.001	79.2 ± 18.3	81.9 ± 19.6	76.9 ± 21.4	0.057	76.6 ± 18.6	79.6 ± 18.9	75.7 ± 18.4	0.031
Heart rate (beats per min)	87.8 ± 24.5	92.3 ± 25.9	96.4 ± 26.5	<0.001	90.9 ± 23.9	95.2 ± 25.5	99.3 ± 24.9	<0.001	87.7 ± 23.6	91.9 ± 27.7	95.6 ± 29.0	0.016	81.7 ± 18.6	85.2 ± 24.1	90.2 ± 27.7	<0.001
**Laboratory findings**																
WBC count (10^9^/L)	7680.1 ± 3631.4	8070.5 ± 3121.2	9891.4 ± 4757.4	<0.001	7850.0 ± 3832.3	8242.2 ± 3217.0	9882.1 ± 5185.0	<0.001	7967.0 ± 3681.9	8132.4 ± 3061.8	10602.2 ± 4602.7	<0.001	7191.5 ± 313.8	7591.6 ± 2860.5	9526.0 ± 3664.7	<0.001
Hemoglobin (mg/dL)	12.7 ± 2.3	12.7 ± 2.4	12.0 ± 2.3	<0.001	13.1 ± 2.2	12.9 ± 2.3	12.3 ± 2.3	<0.001	12.2 ± 2.6	12.5 ± 2.4	11.6 ± 2.1	0.001	12.4 ± 2.1	12.2 ± 2.4	11.7 ± 2.2	<0.001
BUN (mg/dL)	23.3 ± 4.3	25.6 ± 15.8	29.0 ± 18.1	<0.001	23.4 ± 14.0	26.1 ± 16.7	29.9 ± 18.4	<0.001	24.3 ± 16.1	23.9 ± 12.6	29.9 ± 20.0	0.001	23.1 ± 14.7	24.7 ± 14.3	27.8 ± 17.5	<0.001
Creatinine (mg/dL)	1.3 ± 1.3	1.5 ± 1.4	1.6 ± 1.6	<0.001	1.3 ± 1.4	1.5 ± 1.5	1.7 ± 1.5	<0.001	1.4 ± 1.4	1.3 ± 0.9	2.0 ± 2.4	<0.001	1.3 ± 1.2	1.3 ± 1.2	1.6 ± 1.7	<0.001
BNP (pg/ml), *n* = 1,572	963.3 ± 1040.3	1309.0 ± 1181.8	15435.0 ± 1410.4	<0.001	1215.0 ± 1126.6	1571.1 ± 1287.7	1855.8 ± 1594.5	<0.001	664.0 ± 724.2	1177.7 ± 1092.4	1492.2 ± 1135.4	<0.001	689.1 ± 859.0	939.1 ± 893.9	1166.8 ± 1035.2	<0.001
NT-proBNP (pg/ml), *n* = 2,038	6069.9 ± 9110.3	8000.3 ± 9777.0	12560.0 ± 12205.5	<0.001	7020.6 ± 9913.0	9439.5 ± 11003.4	14605.6 ± 13071.4	<0.001	6648.0 ± 9979.7	6314.4 ± 7907.4	11451.5 ± 10863.7	<0.001	4575.9 ± 7456.6	5587.6 ± 6622.9	8942.7 ± 9498.9	<0.001
CRP (mg/dL)	0.16 ± 0.10	0.62 ± 0.22	4.89 ± 4.81	<0.001	0.17 ± 0.10	0.61 ± 0.21	4.56 ± 4.58	<0.001	0.16 ± 0.10	0.63 ± 0.25	5.67 ± 5.16	<0.001	0.16 ± 0.10	0.64 ± 0.22	5.18 ± 5.08	<0.001
**Echocardiographic parameters**																
LVEF (%)	38.9 ± 15.9	37.4 ± 15.4	37.9 ± 15.9	0.050	27.1 ± 7.4	26.9 ± 7.7	26.8 ± 7.9	0.715	44.7 ± 2.6	44.6 ± 2.5	44.7 ± 2.5	0.993	54.6 ± 9.0	53.6 ± 9.1	54.3 ± 9.1	0.199
E/e′	20.5 ± 11.6	21.5 ± 10.7	21.4 ± 11.8	0.102	21.9 ± 12.0	23.0 ± 11.0	22.6 ± 12.3	0.236	19.9 ± 11.5	20.1 ± 9.3	20.3 ± 12.1	0.950	18.9 ± 11.0	19.3 ± 10.0	19.8 ± 11.0	0.425
**Medication**																
Beta-blockers	52.7%	55.5%	50.1%	0.626	57.0%	59.4%	52.3%	0.019	59.1%	60.8%	56.1%	0.660	46.9%	49.5%	46.8%	0.643
ACEi or ARB	69.3%	70.1%	62.4%	<0.001	76.6%	75.4%	68.4%	<0.001	68.7%	70.2%	62.2%	0.230	59.5%	61.8%	53.4%	0.022
MRA	45.5%	48.9%	43.6%	0.028	51.8%	55.3%	48.0%	0.019	35.4%	40.3%	38.3%	0.602	37.1%	39.1%	37.2%	0.769
Statin	55.5%	52.2%	59.4%	0.001	55.6%	52.8%	62.7%	<0.001	65.2%	57.5%	61.1%	0.306	55.4%	51.2%	54.4%	0.373

ACEi, angiotensin-converting enzyme inhibitor; ARB, angiotensin II receptor blocker; BNP, B-type natriuretic peptide; BP, blood pressure; BUN, blood urea nitrogen; COPD, chronic obstructive pulmonary disease; CRP, C-reactive protein; HF, heart failure; eGFR, estimated glomerular filtration rate; HFmrEF, heart failure with mild reduced ejection fraction; HFpEF, heart failure with preserved ejection fraction; HFrEF, heart failure with reduced ejection fraction; LVEF, left ventricular ejection fraction; MRA, mineralocorticoid receptor antagonist; NT-proBNP, N-terminal pro-B-type natriuretic peptide; NYHA, New York Heart Association; WBC, white blood cell.

### In-hospital mortality

**FIGURE 1:**
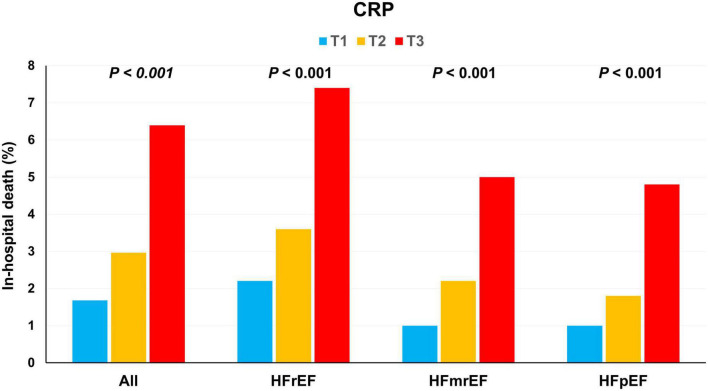
In-hospital mortality according to the C-reactive protein (CRP) tertiles. In-hospital mortality increased with an increase in the CRP tertiles in HFrEF, HFmrEF, HFpEF, and whole population. CRP, C-reactive protein; HFmrEF, heart failure with mild reduced ejection fraction; HFpEF, heart failure with preserved ejection fraction; HFrEF, heart failure with reduced ejection fraction.

Altogether, 139 (3.6%) patients died during the index admission. Mortality was higher among patients with HFrEF than among those with HFmrEF or HFpEF (4.4% vs. 2.7% vs. 2.5%, *P* = 0.013). Among patients with HFrEF, in-hospital mortality increased gradually from T1 to T3 (T1: 2.2, T2: 3.6, and T3: 7.4%; χ^2^ test for linear-by-linear association, *P* < 0.001). Similar findings were observed for HFmrEF and HFpEF or whole population (Figure 1).

In the multivariable analysis, an increase in the CRP tertiles was independently associated with in-hospital outcomes in the whole population [odds ratio (OR) 1.78, 95% CI 1.33–2.39] as well as in the subgroups of HFrEF (OR 1.58, 95% CI 1.09–2.30), HFmrEF (OR 1.51, 95% CI, 0.72–3.52), HFpEF (OR 2.98, 95% CI, 1.46–6.33) (Table 2).

**TABLE 2:** Prognostic value of C-reactive protein (CRP) tertiles according to the heart failure (HF) categories.

	All			HFrEF			HFmrEF			HFpEF		
	OR	95% CI	*P*-value	OR	95% CI	*P*-value	OR	95% CI	*P*-value	OR	95% CI	*P*-value
**In-hospital mortality**												
**Unadjusted**												
For each CRP tertile increase	2.05	1.63–2.57	<0.001	1.94	1.50–2.56	<0.001	2.28	1.17–5.03	0.023	2.32	1.38–4.17	0.002
T1	Reference			Reference			Reference			Reference		
T2	1.80	1.06–3.10	0.031	1.80	1.06–3.10	0.031	2.21	0.43–16.12	0.362	1.75	0.46–7.13	0.407
T3	4.03	2.56–6.58	<0.001	4.03	2.56–6.58	<0.001	5.16	1.31–34.14	0.038	4.93	1.79–17.33	0.005
**Adjusted for GWTG-HF score**												
For each CRP tertile increase	1.84	1.44–2.34	<0.001	1.68	1.26–2.26	<0.001	2.10	1.07–4.62	0.042	2.30	1.33–4.37	0.005
T1	Reference			Reference			Reference			Reference		
T2	1.91	1.09–3.44	0.026	1.73	0.90–3.47	0.107	2.27	0.43–16.55	0.348	2.10	5.08–10.36	0.314
T3	3.43	2.09–5.90	<0.001	2.84	1.58–5.43	<0.001	4.54	1.14–30.24	0.056	5.12	1.66–22.38	0.011
**Adjusted for covariates**												
For each CRP tertile increase	1.78	1.33–2.39	<0.001	1.58	1.09–2.30	0.017	1.51	0.72–3.52	0.297	2.98	1.46–6.73	0.004
T1	Reference			Reference			Reference			Reference		
T2	2.58	1.29–5.16	0.007	2.84	1.17–6.90	0.021	2.70	0.57–27.68	0.203	2.68	0.48–16.89	0.263
T3	4.02	2.12–7.66	<0.001	3.69	1.59–8.55	0.002	3.31	0.53–20.79	0.268	8.62	2.05–48.81	0.006
**Post-discharge mortality**												
**Unadjusted**												
For each CRP tertile increase	1.39	1.30–1.48	<0.001	1.39	1.27–1.52	<0.001	1.55	1.29–1.87	<0.001	1.32	1.16–1.49	<0.001
T1	Reference			Reference			Reference			Reference		
T2	1.34	1.16–1.47	<0.001	1.27	1.05–1.53	0.013	1.22	0.82–1.80	0.328	1.62	1.24–2.11	<0.001
T3	1.92	1.68–2.19	<0.001	1.91	1.60–2.27	<0.001	2.33	1.63–3.33	<0.001	1.75	1.35–2.26	<0.001
**Adjusted for GWTG-HF score**												
For each CRP tertile increase	1.26	1.18–1.35	<0.001	1.22	1.12–1.34	<0.001	1.47	1.23–1.77	<0.001	1.25	1.10–1.41	<0.001
T1	Reference			Reference			Reference			Reference		
T2	1.28	1.11–1.48	<0.001	1.18	0.98–1.43	0.079	1.26	0.85–1.87	0.251	1.56	1.20–2.05	0.001
T3	1.60	1.39–1.83	<0.001	1.49	1.24–1.78	<0.001	2.12	1.48–3.06	<0.001	1.57	1.21–2.04	<0.001
**Adjusted for covariates**												
For each CRP tertile increase	1.22	1.13–1.32	<0.001	1.20	1.08–1.33	<0.001	1.38	1.12–1.70	0.002	1.19	1.02–1.38	0.025
T1	Reference			Reference			Reference			Reference		
T2	1.22	1.04–1.43	0.015	1.13	0.91–1.39	0.264	1.32	0.85–2.03	0.216	1.37	1.02–1.85	0.038
T3	1.49	1.28–1.74	<0.001	1.43	1.17–1.76	0.001	1.90	1.26–2.88	0.001	1.42	1.04–1.92	0.027

The odds and hazard ratios are presented unadjusted, adjusted for GWTG-HF score, and covariates. Regarding covariates, in-hospital mortality was adjusted for age, sex, new-onset heart failure, diabetes, ischemic heart disease, chronic obstructive pulmonary disease, cerebrovascular disease, New York Heart Association class, systolic blood pressure, heart rate, white blood cell count, hemoglobin, blood urea nitrogen, E/e′, left ventricular ejection fraction, use of renin-angiotensin system inhibitors, beta-blockers, and mineralocorticoid receptor antagonists before admission, and CRP tertiles. The post-discharge mortality was adjusted for age, sex, new-onset heart failure, diabetes, ischemic heart disease, chronic obstructive pulmonary disease, cerebrovascular disease, New York Heart Association class, systolic blood pressure, heart rate, white blood cell count, hemoglobin, blood urea nitrogen, E/e′, left ventricular ejection fraction, use of renin-angiotensin system inhibitors, beta-blockers, and mineralocorticoid receptor antagonists at discharge, and CRP tertiles. CI, confidence interval; CRP, C-reactive protein; GWTG-HF, get with the guideline-heart failure; HFmrEF, heart failure with mild reduced ejection fraction; HFpEF, heart failure with preserved ejection fraction; HFrEF, heart failure with reduced ejection fraction; HR, hazard ratio; OR, odds ratio.

Since the WBC count increases during inflammation, we also investigated the prognostic value of WBC count. There was a weak, but significant correlation between WBC count and CRP (*r* = 0.27, *P* < 0.001). WBC count was not associated with increased in-hospital mortality (*P* = 0.118). However, it was associated with post-discharge mortality (*P* < 0.001). There was a weak correlation between CRP and BNP (*r* = 0.11, *P* < 0.001) and between CRP and N-terminal-pro-BNP (*r* = 0.18, *P* < 0.001), suggesting that CRP levels are less dependent on the natriuretic peptide levels.

### Post-discharge mortality

**FIGURE 2:**
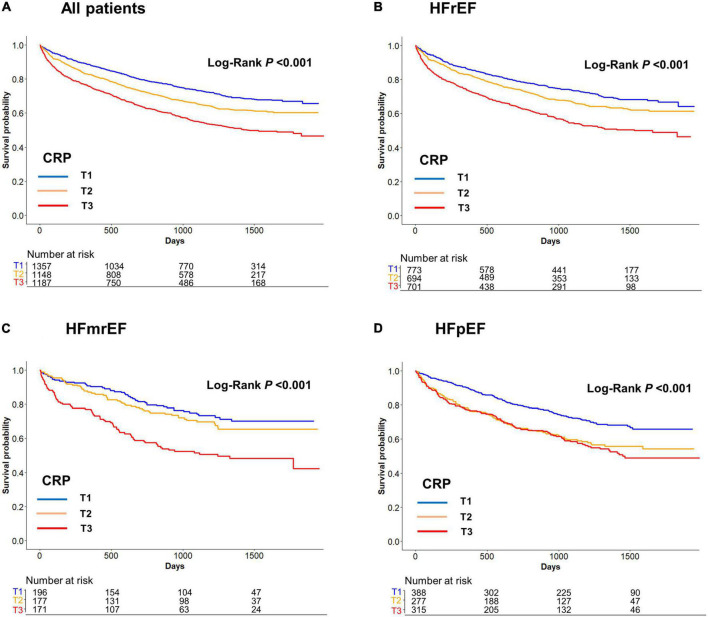
**(A)** All patients, **(B)** HFrEF, **(C)** HFmrEF, and **(D)** HFpEF. Post-discharge mortality according to the C-reactive protein (CRP) tertiles. Post-discharge mortality increased with an increase in the CRP tertiles in HFrEF, HFmrEF, HFpEF, and whole population. CRP, C-reactive protein; HFmrEF, heart failure with mild reduced ejection fraction; HFpEF, heart failure with preserved ejection fraction; HFrEF, heart failure with reduced ejection fraction.

Among 3,692 patients who discharged alive, 1,269 (34.4%) patients died during a median follow-up of 995 days (interquartile range: 365–1,386 days). In contrast to the in-hospital mortality, the post-discharge mortality did not differ between the HFrEF, HFmrEF, and HFpEF groups (34.4% vs. 33.1% vs. 35.1%, *P* = 0.730). After stratification according to the CRP tertiles, there was a gradual increase in mortality with an increase in the CRP tertiles in HFrEF (T1: 27.3, T2: 32.6, T3: 43.9%; *P* < 0.001), HFmrEF (T1: 24.5, T2: 29.4, and T3: 46.8%; *P* < 0.001), and HFpEF (T1: 27.8, T2: 39.0, and T3: 40.6; *P* = 0.001) (Figure 2).

In the Cox proportional hazards regression analysis after adjustment for significant covariates, an increase in the CRP tertiles was independently associated with post-discharge mortality in the whole population [hazard ratio (HR) 1.22, 95% CI 1.13–1.32] as well as in the subgroups of HFrEF (HR 1.20, 95% CI 1.08–1.33), HFmrEF (HR 1.38, 95% CI, 1.12–1.70), and HFpEF (HR 1.19, 95% CI, 1.02–1.38) (Table 2). The results of the sensitivity analysis performed with adjustment for the GWTG-HF score were consistent with those of the main analyses. We also performed sensitivity analysis by classifying HF as HFrEF (LVEF ≤ 40%) and HFpEF (LVEF > 40%) because when KorAHF registry was designed in 2010, the LVEF cutoff for HFpEF was 40% which was also recommended by 2005 ESC-HF guideline. These results by two categories were similar as primary results (Supplementary Figures 1, 2).

### Impact of statins on mortality according to the C-reactive protein tertiles

Among total 3,692 patients who discharged alive, 2,096 (56.8%) patients received statin. Baseline characteristics according to statin use are shown in Supplementary Table 2. Overall, patients with statin-use were more likely to be old and male, and had more co-morbidities than those without statin-use. By multivariable Cox proportional hazard regression models, statin use was not associated with all-cause mortality in the whole population or in patients with HFrEF (Table 3). However, in patients with LVEF > 40% (HFmrEF plus HFpEF), statin users showed better survival trend than those without statin use with a marginal statistical significance. This trend only observed in patients with elevated CRP levels (CRP tertile 1 and 2: HR 0.87, 95% CI 0.69–1.10, *P* = 0.254; tertile 3: HR 0.76, 95% CI 0.56–1.02, *P* = 0.065). Among these patients with LVEF > 40% plus CRP tertile 3, statin use was associated with 46% reduced risk for mortality in those with ischemic heart disease (HR 0.54, 95% CI 0.31–0.97).

**TABLE 3:** Risk of all-cause mortality according to statin use (vs. non-use).

	Adjusted HR*	95% CI	*P*-value
**Whole population (*n* = 3,692)**	0.95	0.84–1.07	0.363
CRP tertile 1 and 2 (*n* = 2,505)	0.90	0.76–1.05	0.136
CRP tertile 3 (*n* = 1,187)	0.99	0.81–1.20	0.902
With IHD (*n* = 1,017)	0.98	0.77–1.26	0.890
Without IHD (*n* = 2,677)	0.93	0.81–1.07	0.288
**HFrEF (*n* = 2,168)**	1.02	0.87–1.20	0.786
CRP tertile 1 and 2 (*n* = 1,467)	0.90	0.73–1.11	0.344
CRP tertile 3 (*n* = 701)	1.11	0.85–1.46	0.439
**HFmrEF + HFpEF (*n* = 1,524)**	0.84	0.70–1.00	0.056
CRP tertile 1 and 2 (*n* = 1,038)	0.87	0.69–1.10	0.254
CRP tertile 3 (*n* = 486)	0.76	0.56–1.02	0.065
- with IHD (*n* = 123)	0.54	0.31–0.97	0.039
- without IHD (*n* = 363)	0.81	0.58–1.14	0.224

*Adjusted for age, sex, diabetes, ischemic heart disease, smoking, and blood urea nitrogen. CI, confidence interval; CRP, C-reactive protein; HFmrEF, heart failure with mild reduced ejection fraction; HFpEF, heart failure with preserved ejection fraction; HFrEF, heart failure with reduced ejection fraction; HR, hazard ratio; IHD, ischemic heart disease.

## Discussion

In the present study, we examined the prognostic value of CRP according to the types of HF. We observed that (i) CRP was an excellent prognostic marker for both HFrEF, HFmrEF, and HFpEF with a similar effect size and (ii) there was a very weak correlation between CRP and natriuretic peptide. Thus, inflammation is important for HF and is independent of the neurohumoral pathway. (iii) Additionally, in only patients with LVEF > 40% with highest CRP tertile, statin-users showed better survival trend than those without statins.

### Comparison of C-reactive protein level and outcomes between heart failure with reduced and preserved ejection fraction

Currently, HF is classified according to EF (31), mainly because patients with similar EF show similar responses to pharmacologic and non-pharmacologic treatments (12). The difference in the cardiac anatomy contributes to the differences in hemodynamic and neurohumoral effects (12). Patients having HFrEF with enlarged LV diameter exhibit higher BNP levels than patients with HFpEF, implying a higher degree of neurohumoral activation (11). In this study the CRP levels were similar between HFrEF, HFmrEF, and HFpEF, although HFmrEF and HFpEF had numerically higher CRP level than HFrEF patients. Previous studies investigating the differential effects of inflammatory markers according to HF phenotypes showed inconsistent results (14–16, 23). For example, Tromp and colleagues showed 96 patients with chronic HFpEF, defined as LVEF ≥ 45%, had higher CRP level than 364 patients with HFrEF, defined as LVEF < 45%. The difference in the study population may have led to different results. However, our study has a large sample size, and the patients had been carefully selected so those with infection as trigger for the acute decompensation were excluded. In this study, CRP seems to be independent of LVEF, since CRP level and the magnitude of its prognostic impact did not differ between HFrEF and HFpEF. This key finding suggests that the neurohumoral and inflammatory pathways are two distinct pathways. The third pathway is the cardiometabolic pathway, which also seems to be independent of LVEF. While drugs that modulated neuro-humoral activation improved outcomes in HFrEF who have higher BNP levels than HFpEF (12), empagliflozin, an SGLT2-inhibitor which mainly acts on cardiometaboilc pathway, improved the outcomes in both HFrEF and HFpEF with similar impact size (18, 19).

We observed a gradual increase in both in-hospital and post-discharge mortality with an increase in the CRP levels, suggesting a dose-dependent relationship. However, the exact mechanisms are still not well defined. It is also unclear whether CRP is a marker or mediator of HF progression. CRP is an acute-phase protein that mediates and perpetuates inflammation and may accelerate the cardiac remodeling process. Interleukin-6 (IL-6) induces CRP production in the liver (32, 33) and IL-1β is a central inflammatory cytokine that drives the IL-6 signaling pathway. Canakinumab is a fully human monoclonal antibody that inhibits IL-1β and has been shown to reduce CRP levels and the composite of death, myocardial infarction, or stroke (34).

### Effect of statins according to the C-reactive protein levels

Another important finding in the present study is that statins seem to be beneficial in patients with elevated CRP levels. In our analysis patients with LVEF > 40% and CRP tertile 3 seem to benefit from statins. Among these patients, statin use was associated with 44% reduced risk for mortality in those with ischemic heart disease. Although statins have robustly improved outcomes in high-risk patients (35), their effect seems to be neutral in patients with HF. In the Controlled Rosuvastatin Multinational Trial in Heart Failure (25) and the GISSI-HF study (26), statins did not improve the outcomes in patients with HF. Nevertheless, in the Justification for the Use of Statins in Primary Prevention study (24), the use of 20 mg of rosuvastatin in patients with CRP ≥ 2.0 mg/dL reduced the CRP levels by 37% and the composite of myocardial infarction, stroke, arterial revascularization, hospitalization for unstable angina, or death from cardiovascular causes by 46%. It is well known that not all patients benefit equally from a specific therapy. For example, the effect of beta-blockers seems to be greater in patients having HFrEF with a higher heart rate (36). Similarly, statins that modulate inflammation may be more effective in patients having HF with elevated CRP levels, as shown in the present study. This hypothesis should be evaluated in clinical trials.

### Limitations

The present study has several limitations. Although we adjusted for significant clinical factors and the GWTG-HF score, there might be residual confounding factors that could have influenced the relationship between CRP levels and clinical outcomes due to the observational nature of the study. In patients with stable HF, the CRP level remains relatively stable (24). Therefore, we excluded patients whose acute decompensation was triggered by infection. Although data regarding the triggers in each patient were prospectively collected, confirmed, and adjudicated by the investigators, their interpretation remains subjective and was based on the judgment of the investigators, which might limit the reproducibility of the study results. Since we enrolled only the patients hospitalized for acute HF in East Asia, the generalizability of our results to other racial groups and to patients with chronic stable HF might be limited. Finally, the number of patients with HFmrEF was relatively small (*n* = 559), thus predisposes to type II error, i.e., false negative results.

## Conclusion

In patients with acute HF whose decompensation was not triggered by infection, CRP was an excellent prognostic marker for both HFrEF and HFpEF with a similar effect size. Additionally, there was no significant correlation between CRP levels and LVEF and a weak correlation between CRP levels and natriuretic peptide, suggesting that the neurohumoral and inflammatory pathways might be two independent pathways for the progression of HF. We observed that only the patients with high CRP levels benefited from statins. Designated clinical trials are necessary to address whether statins may have a differential effect on the degree of inflammation.

## Data availability statement

The data generated in this study is available from the corresponding author upon reasonable request. Requests to access these datasets should be directed to D-JC, djchoi@snubh.org.

## Ethics statement

The studies involving human participants were reviewed and approved by the Seoul National University Bundang Hospital, IRB No. B-1104-125-014. The patients/participants provided their written informed consent to participate in this study.

## Author contributions

JP and D-JC contributed to the conception and design of the work. JP, MY, and D-JC drafted the manuscript and contributed to the review and revision of the manuscript. H-WC, H-JC, KK, DY, B-SY, S-MK, SB, E-SJ, J-JK, M-CC, SC, and B-HO assisted in data collection and analysis. All authors contributed to the article and approved the submitted version.
