# Supplementary material for: C-reactive protein and statins in heart failure with reduced and preserved ejection fraction
Source: Front Cardiovasc Med. 2022 Dec 23;9:1064967. doi: 10.3389/fcvm.2022.1064967 (PMC9816146; doi:10.3389/fcvm.2022.1064967)
Supplement: Supplementary file 1 [file Data_Sheet_1.docx]

**Supplementary Table 1. Baseline characteristics of patients included and excluded due to missing CRP levels or LVEF data**

|  | Included  (n=3,831) | Excluded  (n=692) | P-value |
| --- | --- | --- | --- |
| Age (years) | 68.2±14.4 | 68.5±14.3 | 0.626 |
| Men (%) | 53.9% | 53.0% | 0.713 |
| De-novo (%) | 55.5% | 41.9% | <0.001 |
| Body mass index (kg/m^2^) | 23.4±3.8 | 23.5±4.2 | 0.396 |
| **Past Medical history** |  |  |  |
| Hypertension (%) | 58.8% | 59.5% | 0.742 |
| Diabetes mellitus (%) | 35.0% | 35.5% | 0.805 |
| eGFR <60 ml/min/1.73m^2^ (%) | 43.6% | 50.3% | 0.001 |
| Ischemic heart disease (%) | 72.2% | 70.5% | 0.382 |
| Atrial fibrillation (%) | 26.8% | 36.8% | <0.001 |
| COPD (%) | 10.6% | 9.7% | 0.497 |
| Cerebrovascular disease (%) | 14.8% | 16.3% | 0.312 |
| Malignancy (%) | 7.9% | 10.3% | 0.049 |
| Current smoking (%) | 18.4% | 15.2% | 0.047 |
| NYHA functional class (%) |  |  | 0.006 |
| II | 16.4% | 15.5% |  |
| III | 36.6% | 42.9% |  |
| IV | 47.0% | 41.6% |  |
| **Physical Exam** |  |  |  |
| Systolic BP (mmHg) | 131.7±29.9 | 124.9±30.6 | <0.001 |
| Diastolic BP (mmHg) | 78.9±18.7 | 76.3±18.1 | 0.001 |
| Heart rate (beats per min) | 92.1±25.9 | 91.4±28.0 | 0.554 |
| **Laboratory findings** |  |  |  |
| WBC count (10^9^/L) | 8532.3±4022.5 | 8300.2±4134.2 | 0.165 |
| Hemoglobin (mg/dL) | 12.5±2.3 | 12.5±2.4 | 0.753 |
| BUN (mg/dL) | 25.9±16.3 | 27.9±17.8 | 0.005 |
| Creatinine (mg/dL) | 1.5±1.4 | 1.6±1.8 | 0.024 |
| BNP (pg/mL) | 1264.0±1240.4 | 1390.7±1422.5 | 0.254 |
| NT-proBNP (pg/mL) | 8841.9±10790.2 | 8678.3±10160.5 | 0.777 |
| CRP (mg/dL), | 1.88±3.52 | 2.01±3.80* | 0.425 |
| **Echocardiographic parameters** |  |  |  |
| LVEF (%) | 38.1±15.8 | 34.4±15.5* | <0.001 |
| E/e’ | 21.1±11.4 | 21.0±12.5 | 0.855 |
| **Medication** |  |  |  |
| Beta-blockers | 52.7% | 40.9% | <0.001 |
| ACEi or ARB | 67.2% | 58.5% | <0.001 |
| MRA | 45.9% | 42.3% | 0.087 |
| Statin | 55.8% | 50.6% | 0.013 |

*CRP level (n=522 for excluded patients), LVEF data (n=373 for excluded patients)

ACEi, angiotensin-converting enzyme inhibitor; ARB, angiotensin II receptor blocker; BNP, B-type natriuretic peptide; BP, blood pressure; BUN, blood urea nitrogen; COPD, chronic obstructive pulmonary disease; CRP, C-reactive protein; HF, heart failure; eGFR, estimated glomerular filtration rate; HFpEF, heart failure with preserved ejection fraction; HFrEF, heart failure with reduced ejection fraction; LVEF, left ventricular ejection fraction; MRA, mineralocorticoid receptor antagonist; NT-proBNP, N-terminal pro-B-type natriuretic peptide; NYHA, New York Heart Association; WBC, white blood cell.

**Supplementary Table 2. Baseline characteristics of patients according to statin use**

|  | Statin use  (n=2,096) | Non-user  (n=1,596) | P-value |
| --- | --- | --- | --- |
| Age (years) | 70.7±12.5 | 64.7±15.9 | <0.001 |
| Men (%) | 56.6% | 49.9% | <0.001 |
| De-novo (%) | 58.1% | 52.3% | 0.001 |
| Body mass index (kg/m^2^) | 23.3±3.6 | 23.5±4.1 | 0.142 |
| **Past Medical history** |  |  |  |
| Hypertension (%) | 66.2% | 48.7% | <0.001 |
| Diabetes mellitus (%) | 43.5% | 23.9% | <0.001 |
| eGFR <60 ml/min/1.73m^2^ (%) | 46.7% | 37.9% | <0.001 |
| Ischemic heart disease (%) | 39.9% | 11.2% | <0.001 |
| Atrial fibrillation (%) | 21.6% | 34.0% | <0.001 |
| COPD (%) | 10.2% | 10.9% | 0.535 |
| Cerebrovascular disease (%) | 15.3% | 13.9% | 0.233 |
| Malignancy (%) | 6.7% | 9.5% | 0.002 |
| Current smoking (%) | 20.1% | 16.2% | 0.002 |
| NYHA functional class (%) |  |  | <0.001 |
| II | 14.5% | 19.5% |  |
| III | 35.7% | 39.3% |  |
| IV | 49.8% | 412% |  |
| **Physical Exam** |  |  |  |
| Systolic BP (mmHg) | 135.2±30.2 | 128.5±29.0 | <0.001 |
| Diastolic BP (mmHg) | 79.5±17.9 | 78.9±19.6 | 0.352 |
| Heart rate (beats per min) | 91.9±24.9 | 92.2±26.9 | 0.661 |
| **Laboratory findings** |  |  |  |
| WBC count (10^9^/L) | 8913.4±4261.1 | 7903.2±3408.8 | <0.001 |
| Hemoglobin (mg/dL) | 12.4±2.2 | 12.7±2.4 | <0.001 |
| BUN (mg/dL) | 25.9±15.5 | 25.1±16.4 | 0.122 |
| Creatinine (mg/dL) | 1.5±1.5 | 1.3±1.3 | <0.001 |
| BNP (pg/mL), n=1,517 | 1293.9±1282.9 | 1188.3±1178.6 | 0.096 |
| NT-proBNP (pg/mL), n=1,969 | 9430.1±10815.1 | 7607.1±10528.9 | <0.001 |
| CRP (mg/dL) | 2.00±3.70 | 1.51±2.84 | <0.001 |
| **Echocardiographic parameters** |  |  |  |
| LVEF (%) | 38.1±14.6 | 38.6±17.0 | 0.371 |
| E/e’ | 21.1±11.0 | 21.0±12.0 | 0.814 |
| **Medication** |  |  |  |
| Beta-blockers | 60.0% | 47.4% | <0.001 |
| ACEi or ARB | 72.6% | 65.3% | <0.001 |
| MRA | 45.8% | 48.9% | 0.069 |

ACEi, angiotensin-converting enzyme inhibitor; ARB, angiotensin II receptor blocker; BNP, B-type natriuretic peptide; BP, blood pressure; BUN, blood urea nitrogen; COPD, chronic obstructive pulmonary disease; CRP, C-reactive protein; HF, heart failure; eGFR, estimated glomerular filtration rate; HFpEF, heart failure with preserved ejection fraction; HFrEF, heart failure with reduced ejection fraction; LVEF, left ventricular ejection fraction; MRA, mineralocorticoid receptor antagonist; NT-proBNP, N-terminal pro-B-type natriuretic peptide; NYHA, New York Heart Association; WBC, white blood cell.

**Supplementary Figure 1. In-hospital mortality according to CRP tertiles in heart failure with reduced and preserved ejection fraction.**
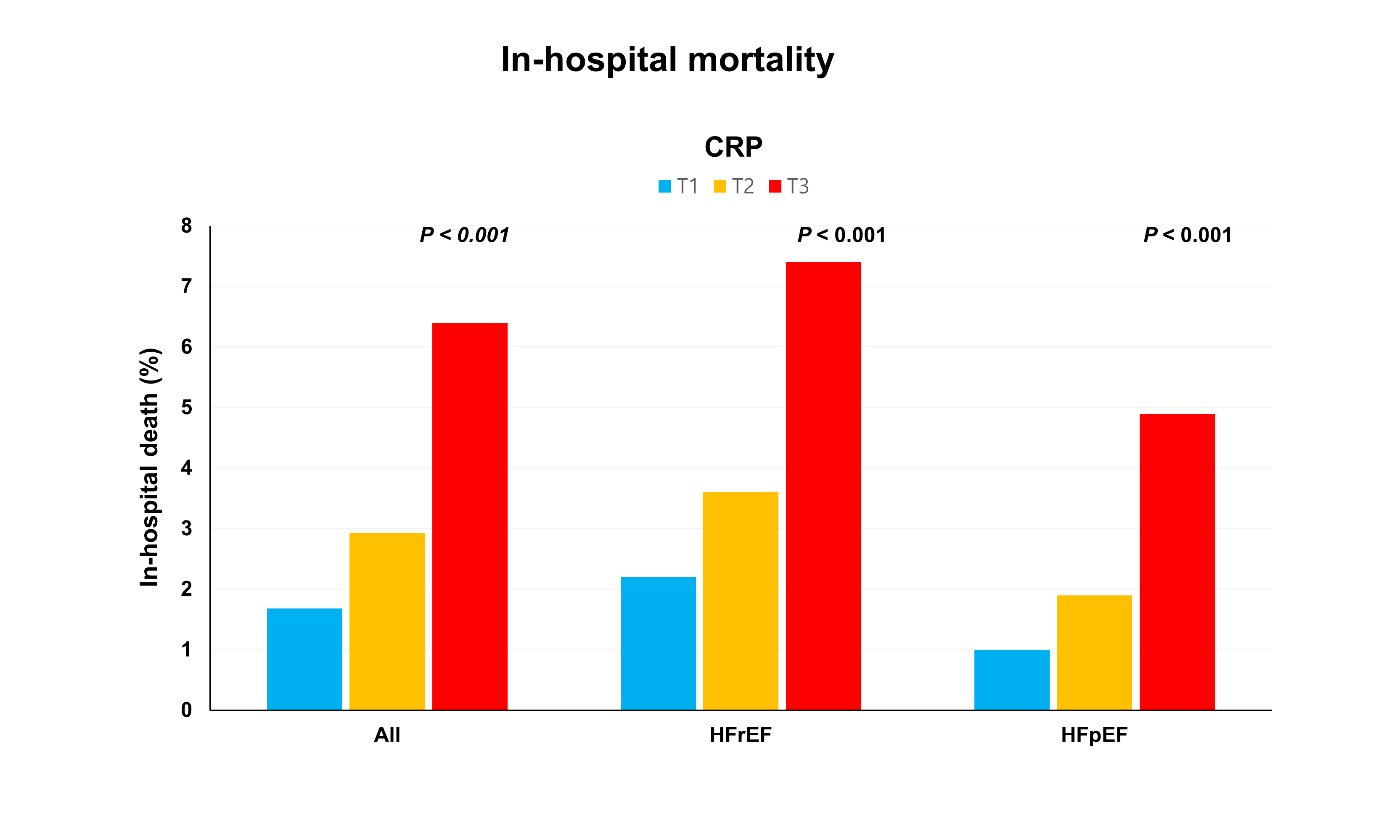


In-hospital mortality increased with an increase in the CRP tertiles in HFrEF (LVEF ≤40%) as well as in HFpEF (LVEF >40%).

C-reactive protein, CRP; Heart failure with preserved ejection fraction, HFpEF; Heart failure with reduced ejection fraction, HFrEF. LVEF, left ventricular ejection fraction.

**Supplementary Figure 2. Post-discharge mortality according to the CRP tertiles in heart failure with reduced and preserved ejection fraction.**

**
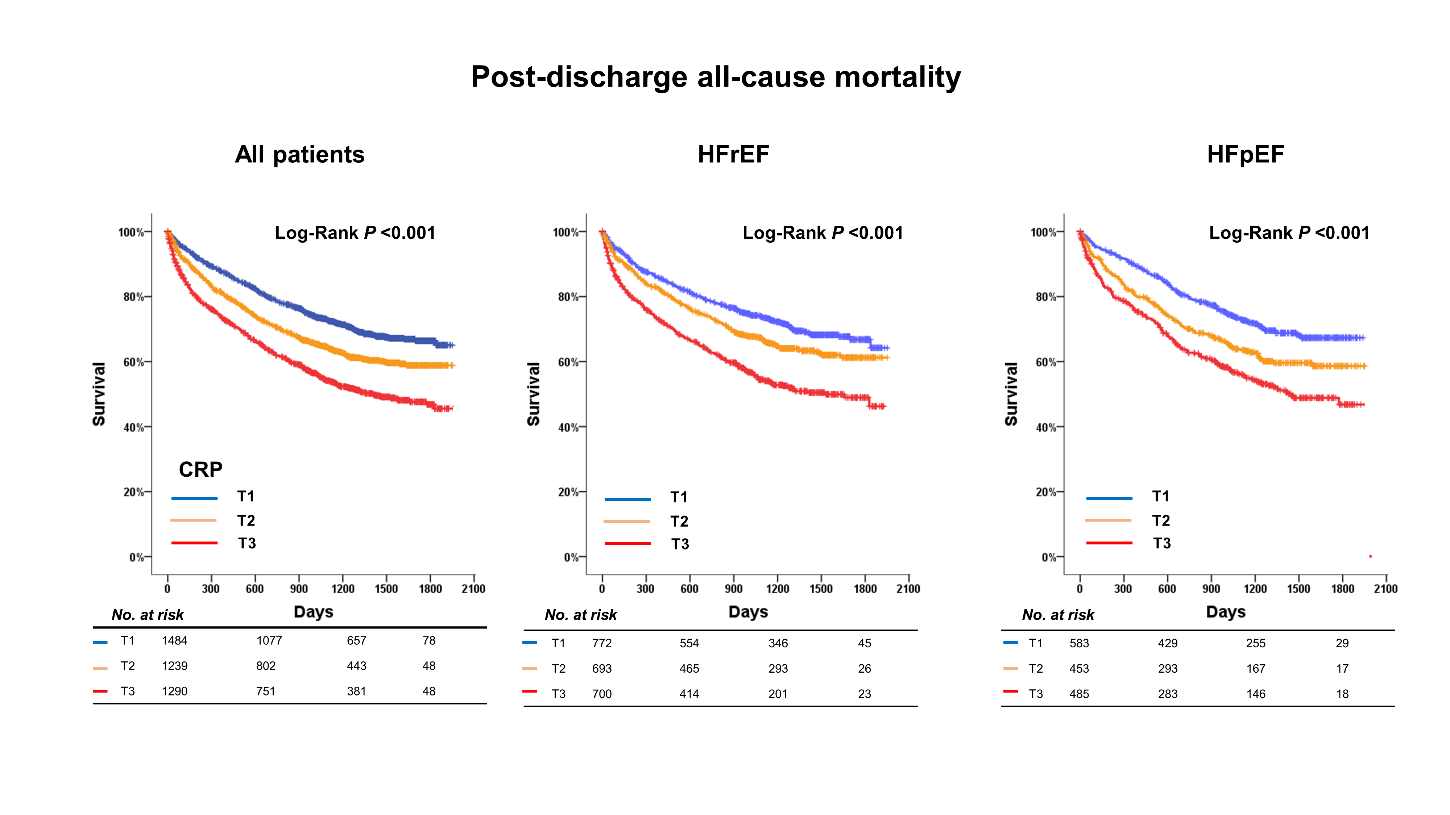
**

Post-discharge mortality increased with an increase in the CRP tertiles in HFrEF (LVEF ≤40%) as well as in HFpEF (LVEF >40%).

C-reactive protein, CRP; Heart failure with preserved ejection fraction, HFpEF; Heart failure with reduced ejection fraction, HFrEF; LVEF, left ventricular ejection fraction.
